# Clustering cancers by shared transcriptional risk reveals novel targets for cancer therapy

**DOI:** 10.1186/s12943-022-01592-y

**Published:** 2022-05-18

**Authors:** Hua Gao, Richard A. Baylis, Lingfeng Luo, Yoko Kojima, Caitlin F. Bell, Elsie G. Ross, Fudi Wang, Nicholas J. Leeper

**Affiliations:** 1 Division of Vascular Surgery, Department of Surgery, Stanford University School of Medicine, Stanford, CA 94305 USA; 2 Stanford Cardiovascular Institute, Stanford University, Stanford, CA 94305 USA; 3 Division of Cardiovascular Medicine, Department of Medicine, Stanford University School of Medicine, Biomedical Innovations Building, 240 Pasteur Drive, #3654, Stanford, CA 94305 USA

## Main text

The pursuit of targeted cancer therapies has greatly benefitted from the existence of large transcriptomic datasets, such as The Cancer Genome Atlas (TCGA), which have enabled the correlation of intra-tumoral gene expression with patient survival. Here, we use pathway enrichment data to identify three distinct groups of cancers characterized by cluster-specific biology and diverging mortality rates. To explore the clinical actionability of these findings, we leveraged the drug prediction algorithm, OCTAD [1] to: (1) determine whether any promising investigational drugs can reverse these detrimental gene expression patterns; and (2) ascertain whether any FDA-approved drugs could be repurposed to improve cluster-specific cancer outcomes.

To perform these studies, tumor tissue mRNA-Seq data, patients’ demographic information, and survival status for 27 individual cancer types from the TCGA [2] (updated through May 2021) were used to compute a survival analysis for each gene and each cancer type. We then used each gene’s correlation with patient outcomes to perform a GSEA [3] hallmark pathway enrichment analysis [4], allowing us to understand how each of the pathways correlated with patient survival for each cancer type. Cancers were then clustered using a shared nearest neighbor modularity optimization with the Seurat package [5], as described in the online Methods.

We next used transcriptomic data from the drug disturbance dataset, LINCS [6] (which has disturbance expression data from 71 cell lines treated with 12,442 compounds) to screen for drugs that could reverse the deleterious expression profile associated with each cancer cluster. These data were used to calculate a Kolmogorov-Smirnov statistic [1] to predict the reversal ability of each compound for each cancer type. Briefly, if a compound completely reversed the risk-associated genes (e.g. the mortality-linked (hazard ratio > 1) genes clustered at the downregulated tail of the disturbance expression distribution, as detailed in the online Methods), the reversal score would approach the minimum value of − 1. The cluster-level effect was then defined as the aggregate of the most significant reversal effects for all cancer types belonging to a given cancer cluster. Two strategies were subsequently used to validate the predicted drugs. First, cancer cell lines were treated with the compound predicted to have the strongest cluster-specific beneficial effect, and then submitted for RNA sequencing. The observed reversal score was calculated as the weighted sum of the differential expression of the top 200 detrimental genes, with the survival risk as the weight. Second, a pharmacovigilance study [7] was performed for the FDA-approved drug predicted to have the strongest cluster-specific benefit (restricted to drugs prescribed to > 1000 patients in the Stanford Hospitals). Specifically, 1:5 propensity score matched cohorts (matched on demographics, smoking status, comorbid conditions, procedures, and therapeutics in the 6 months leading up to enrollment) treated with or without the drug of interest were evaluated for cluster-specific cancer incidence within 5 years.

**Fig. 1:**
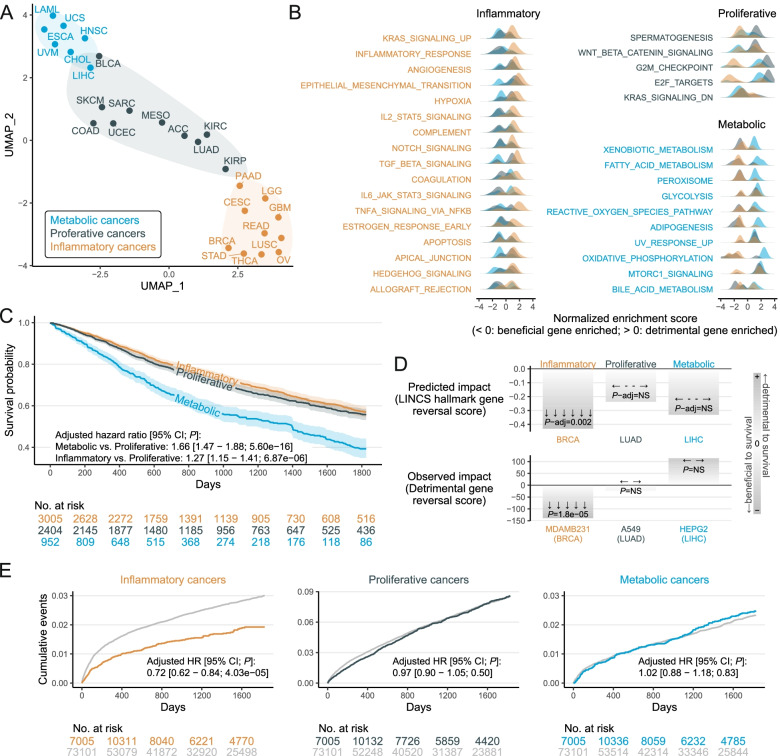
Unbiased genetic analyses identify three distinct cancer clusters which may be targetable in a cluster-specific manner. **A**. Dimensional reduction and clustering of cancer types (full names provided in Table S1) based on transcriptional hallmark pathway expression and correlation with patient survival identifies three cancer subpopulations. **B**. Summary of the detrimental genetic pathways enriched in the ‘inflammatory cluster’ (orange), the ‘metabolic cluster’ (blue), and the ‘proliferative cluster’ (black). **C**. 5-year overall KM survival curves for patients assigned to each cluster. **D**. Drug prediction statistics for the leading compound, AZ-628, which is predicted to specifically rescue the deleterious gene expression profile associated with inflammatory cancers (top subpanel). In vitro validation statistics (reversal score) for AZ-628 demonstrates benefit in a representative inflammatory breast cancer cell line (MDA-MB-231), but no impact on a representative proliferative lung cancer cell line (A549), nor a representative metabolic hepatocellular cancer cell line (HepG2, bottom subpanel). **E**. Propensity-matched pharmacovigilance studies (matched on demographics, smoking status, comorbid conditions, procedures, and therapeutics in the 6 months leading up to enrollment) demonstrate the 5-year incidence of each cancer cluster amongst individuals prescribed clopidogrel, an FDA-approved drug predicted to specifically reduce inflammatory cancers

In contrast to prior reports that focused on a cell-of-origin pattern [2], our pathway-based transcriptomic-survival analysis (Table S2) identified three cancer clusters (Fig. 1A) which had no discernable connection between the cancers that clustered together (e.g., cellular origin, organ system, sex-specific cancers). For example, rectal adenocarcinoma and colon adenocarcinoma were clustered in different groups.

One cluster, which included glioblastoma multiforme and breast cancer, was dominated by inflammatory pathways (Fig. 1B), including cytokine and complement cascades. This suggested that dysregulation of these pathways was associated with worse patient outcomes for these cancers and that targeting these pathways may be uniquely beneficial for these cancers. The second cluster, which included acute myeloid leukemia and hepatocellular carcinoma, was enriched in metabolic pathways like fatty acid metabolism and glycolysis. The remaining cancers, including melanoma and colon adenocarcinoma, were enriched in proliferative pathways, like the G2M checkpoint. Interestingly, when plotted on Kaplan-Meier curves, the metabolic cluster had significantly worse survival (Fig. 1C; hazard ratio (HR) 1.33 vs. inflammatory cancers, *P* < 0.001; HR 1.66 vs. proliferative cancers, *P* < 0.001).

To investigate the clinical relevance of these findings, we then applied the in silico drug repurposing pipeline outlined above. This approach identified numerous preclinical and FDA-approved compounds predicted to effectively reverse the high-risk transcriptional signatures associated with each cancer cluster. Proof-of-principle testing was performed for the top preclinical compound (Table S3), AZ-628 (an experimental Raf inhibitor), and the top FDA-approved drug, clopidogrel (a widely-prescribed antiplatelet medicine). As predicted, 0.1 μM AZ-628 selectively reversed the expression of survival risk genes in vitro in an inflammatory cancer cell line (Fig. 1D). Similarly, clopidogrel use (75 mg/day) amongst ‘real-world’ patients was associated with a specific reduction in the incidence of inflammatory cancers, but had no effect on other cancer types (Fig. 1E, HR 0.72, *P* < 0.001).

## Conclusions

To date, no study has integrated gene-to-survival correlation data to simultaneously identify similarities between cancers and determine which pathways are most important for patient outcomes. While prior multi-omics efforts identified a cell-of-origin pattern across diverse cancer types [2], our analyses demonstrated that malignancies may be better distinguished according to dysregulation of key inflammatory, metabolic, or proliferative pathways. This approach allowed groups of cancers to be stratified by mortality risk, and revealed important biologic similarities that could provide novel mechanistic insights. From a translational perspective, these efforts also identified novel targets that could provide survival benefit for certain cancer types, but may need to be avoided for others. While prospective validation studies are required, this proof-of-principle study shows the potential of integrating tumor transcriptomics and patient survival data to identify important patterns between cancers and predict targets for future cancer therapeutics.

## Supplementary Information

**Additional file 1.**

**Additional file 2.**

## Data Availability

Original RNA-Seq data are available in the NCBI BioProject PRJNA807725.

## Abbreviations

TCGA: The Cancer Genome Atlas
OCTAD: Open Cancer TherApeutic Discovery
GSEA: Gene Set Enrichment Analysis
LINCS: The Library of Integrated Network-Based Cellular Signatures
HR: Hazard ratio
ACC: Adrenocortical carcinoma
BLCA: Bladder Urothelial Carcinoma
BRCA: Breast invasive carcinoma
CESC: Cervical squamous cell carcinoma
CHOL: Cholangiocarcinoma
COAD: Colon adenocarcinoma
ESCA: Esophageal carcinoma
GBM: Glioblastoma multiforme
HNSC: Head and Neck squamous cell carcinoma
KIRC: Kidney renal clear cell carcinoma
KIRP: Kidney renal papillary cell carcinoma
LAML: Acute Myeloid Leukemia
LGG: Brain Lower Grade Glioma
LIHC: Liver hepatocellular carcinoma
LUAD: Lung adenocarcinoma
LUSC: Lung squamous cell carcinoma
MESO: Mesothelioma
OV: Ovarian serous cystadenocarcinoma
PAAD: Pancreatic adenocarcinoma
READ: Rectum adenocarcinoma
SARC: Sarcoma
SKCM: Skin Cutaneous Melanoma
STAD: Stomach adenocarcinoma
THCA: Thyroid carcinoma
UCEC: Uterine Corpus Endometrial Carcinoma
UCS: Uterine Carcinosarcoma
UVM: Uveal Melanoma
