# Supplementary material for: Clustering cancers by shared transcriptional risk reveals novel targets for cancer therapy
Source: Mol Cancer. 2022 May 18;21:116. doi: 10.1186/s12943-022-01592-y (PMC9115915; doi:10.1186/s12943-022-01592-y)
Supplement: Supplementary file 2 — Additional file 2. [file 12943_2022_1592_MOESM2_ESM.docx]

**Data collection**

TCGA Pan-Cancer [1] tumor tissue gene expression profiles were downloaded from UCSC Xena[2] (https://xenabrowser.net/datapages/?cohort=GDC%20Pan-Cancer%20(PANCAN)). The mRNA expression level of each gene was then normalized to the upper quartile (FPKM-UQ). This Pan-Cancer mRNA-Seq dataset covered 33 cancer types as annotated by the TCGA (https://gdc.cancer.gov/resources-tcga-users/tcga-code-tables/tcga-study-abbreviations). The demographic information and follow-up records (through May 6, 2021) were retrieved using the R TCGAbiolinks package[3]. The RNASeq data for CCLE cell lines from BioProject PRJNA523380[4] was downloaded and processed using the same pipeline from TCGA (STAR-2.6.0c 2-pass approach[5] with Gencode v22 transcriptome, and HTSeq-0.6.1p1[6]).

**Two convolution layers: survival analysis and gene-set enrichment analysis**

First, those cancer types with less than 10 death events were removed, including TGCT, DLBC, PCPG, KICH, PRAD, and THYM. Then, for each cancer type and each gene, multivariate Cox regressions were performed using the R survival package[7], with sex and age at index as covariates. The log hazard ratio values were retrieved as the input for the next layer. Then, for each type of cancer, a hallmark gene-set[8] enrichment analysis[9] was performed using the R clusterProfiler package[10]. The normalized enrichment scores were retrieved as the outcome.

**Clustering**

The SNN (shared nearest neighbor) clustering algorithm from R Seurat package[11] was used to identify potential cancer clusters, based on the normalized enrichment score matrix for each cancer type, with parameters npcs = 20, n.neighbors = 5, k.param = 5, and resolution = 1.0. To identify the specific pathways associated with each cluster of cancer types, a multinomial logistic regression model was fitted using the R nnet package `multinom` function, and then the probability of association was predicted assuming the NES = 4. Those pathways with a probability higher than 80% were identified as the pathways specific to each individual cluster.

**In-silico drug screening**

The original OCTAD algorithm[12] supports the input of differential gene expression data from case-control studies. A modified version of the gene expression reversal score calculation was introduced to support the survival risk profile utilized in this study. In short, we examined the LINCS dataset[13] which provides gene disturbance expression profiles for 71 cell lines treated with 12,442 compounds under a variety of conditions. To predict the effect of a given drug on each cancer, we first calculated the reversal gene expression scores (RGES) for all available cell lines belonging to that cancer type and then chose the most significant one as the representative result. A similar strategy was adopted to aggregate the predictions from each cancer type and apply them to each cancer cluster. The RGES was defined as the difference of two distribution similarity statistics (Kolmogorov–Smirnov [KS] statistics) generated by comparing the survival risk profile against the LINCS gene disturbance expression profile. One KS was generated for the detrimental genes (hazard ratio > 1), and another KS was generated for the beneficial genes (hazard ratio < 1). The ideal case would be that the most detrimental genes were inhibited by the compound (i.e. the detrimental genes clustered to the downregulated tail of the drug disturbance expression profiles; KS -> -1), while the most beneficial genes were upregulated by the compound (and would cluster to the upregulated tail of the drug disturbance profile; KS -> 1), leading to an ideal final reversal gene expression score (RGES) approaching -2. The empirical *P*-value was estimated through a 10,000-repeat permutation procedure. This involved: 1. Permutating the drug disturbance expression profile, re-calculating the RGES, and repeating this procedure 10000 times to get 10000 random RGES as the background; 2. Fitting a mixture (across the three clusters) to achieve a normal distribution; and 3. Calculating the lower tail density of the observed RGES.

**In vitro validation of OCTAD prediction through RNASeq**

Because compound AZ-628 was the top cancer cluster-specific drug identified by the preceding in silico prediction studies, it was selected for in vitro validation. One cancer cell line per cancer cluster was chosen according to predictions from the LINCS dataset. MDAMB231 was selected as a breast cancer line representative of inflammatory cancers, A549 was selected as a lung adenocarcinoma line representative of the proliferative cancers, and HEPG2 was selected as a hepatocellular carcinoma line representative of the metabolic cancers. DMEM, RPMI-1640, or EMEM containing 10% FBS, penicillin, and streptomycin was used to culture MDAMB231, A549, or HepG2 cell line, respectively. Those cell lines were treated with AZ-628 or DMSO at a dose of 0.1 μM for 24 hours, and then collected for RNASeq. The RNASeq data was processed using the same pipeline from TCGA[1]. Based on the hazard ratio value for the cancer type and the basal expression level of the cell line, the cell line-specific risk was inferred. The cell line specific risk was then taken as a weight to calculate a weighted sum (reversal gene expression score) of the differential expression of the top 200 detrimental genes. The top 200 genes were determined based on signal-to-noise trade-off (we observed an elbow of the reverse gene expression score curve around the top 200 genes, indicating an unsaturated signal when using less than the top 200 genes, but too much noise when using too many small impact genes).

**Cell cycle analysis with propidium iodide**

Cancer cell lines were treated with 0.1 μM of AZ-628 or DMSO for 24 hours before being harvested and stained with propidium iodide (0.05 mg/mL). Then the cell cycle was analyzed by flow cytometry.

**BrdU incorporation assay**

Different cell lines were treated with 0.1 μM of AZ-628 or DMSO for 24 hours. 3 hours before the endpoint of treatment, 10 μM of BrdU was added to the culture medium. Then, harvested cells were stained following the protocol of eBioscience™ BrdU Staining Kit for Flow Cytometry FITC (catalog number 8811-6600) and analyzed by flow cytometry.

**Annexin V cell apoptosis assay**

0.1 μM of AZ-628 or DMSO was used to treat cells for 24 hours before cell harvesting. Then the samples were stained using the Annexin V Apoptosis Detect Kit FITC (catalog number 8811-6600-42) and analyzed by flow cytometry.

**Drug validation through Stanford electronic medical record**

Because clopidogrel was identified as the top FDA-approved (clinically-available) cancer cluster-specific drug from the in-silico prediction procedure described above, it was selected for the ‘real-world’ validation studies provided in panel E. Clopidogrel generally is prescribed to patients diagnosed with cardiovascular events, such as myocardial infarction, at a dose of 75 mg/day. Thus, those indications were defined as the entry events. Patients prescribed clopidogrel were then defined as being in the treated cohort, while those that were not prescribed this drug were defined as being in the control cohort. All demographic variables (including sex, age, and race), smoking status, comorbid conditions, clinical care procedures and therapeutics in the 6 months leading up to enrollment were collected and modeled into a propensity score matching algorithm (R MatchIt package) to create 1:5 matched cohorts. All the clinical information was retrieved through Stanford STARR de-identified pre-IRB database. Kaplan-Meier analyses and adjusted Cox regressions were used to evaluate cancer incidence within 5 years according to cancer cluster subtype, where clopidogrel usage was treated as the time-dependent variable.

**Reference**

1. Hoadley KA, Yau C, Hinoue T, Wolf DM, Lazar AJ, Drill E, et al. Cell-of-Origin Patterns Dominate the Molecular Classification of 10,000 Tumors from 33 Types of Cancer. Cell. 2018;173:291-304.e6.

2. Goldman MJ, Craft B, Hastie M, Repečka K, McDade F, Kamath A, et al. Visualizing and interpreting cancer genomics data via the Xena platform. Nat Biotechnol. 2020;38:675–8.

3. Colaprico A, Silva TC, Olsen C, Garofano L, Cava C, Garolini D, et al. TCGAbiolinks: an R/Bioconductor package for integrative analysis of TCGA data. Nucleic Acids Research. 2016;44:e71–e71.

4. Ghandi M, Huang FW, Jané-Valbuena J, Kryukov GV, Lo CC, McDonald ER, et al. Next-generation characterization of the Cancer Cell Line Encyclopedia. Nature. 2019;569:503–8.

5. Dobin A, Gingeras TR. Mapping RNA‐seq Reads with STAR. Current Protocols in Bioinformatics [Internet]. 2015 [cited 2021 Nov 22];51. Available from: https://onlinelibrary.wiley.com/doi/10.1002/0471250953.bi1114s51

6. Anders S, Pyl PT, Huber W. HTSeq--a Python framework to work with high-throughput sequencing data. Bioinformatics. 2015;31:166–9.

7. Therneau TM. A Package for Survival Analysis in R [Internet]. 2021. Available from: https://CRAN.R-project.org/package=survival

8. Liberzon A, Birger C, Thorvaldsdóttir H, Ghandi M, Mesirov JP, Tamayo P. The Molecular Signatures Database Hallmark Gene Set Collection. Cell Systems. 2015;1:417–25.

9. Subramanian A, Tamayo P, Mootha VK, Mukherjee S, Ebert BL, Gillette MA, et al. Gene set enrichment analysis: A knowledge-based approach for interpreting genome-wide expression profiles. Proceedings of the National Academy of Sciences. 2005;102:15545–50.

10. Wu T, Hu E, Xu S, Chen M, Guo P, Dai Z, et al. clusterProfiler 4.0: A universal enrichment tool for interpreting omics data. The Innovation. 2021;2:100141.

11. Hao Y, Hao S, Andersen-Nissen E, Mauck WM, Zheng S, Butler A, et al. Integrated analysis of multimodal single-cell data. Cell. 2021;184:3573-3587.e29.

12. Zeng B, Glicksberg BS, Newbury P, Chekalin E, Xing J, Liu K, et al. OCTAD: an open workspace for virtually screening therapeutics targeting precise cancer patient groups using gene expression features. Nat Protoc. 2021;16:728–53.

13. Stathias V, Turner J, Koleti A, Vidovic D, Cooper D, Fazel-Najafabadi M, et al. LINCS Data Portal 2.0: next generation access point for perturbation-response signatures. Nucleic Acids Res. 2020;48:D431–9.
